# Efficient Bioproduction of *p*-Hydroxybenzaldehyde *β*-Glucoside from *p*-Hydroxybenzaldehyde by Glycosyltransferase Mutant UGT_BL_1-Δ60

**DOI:** 10.3390/biology14101358

**Published:** 2025-10-03

**Authors:** Bo Fan, Shunuan Fu, Yijun Zhu, Wei Tang, Yucai He

**Affiliations:** 1 School of Pharmacy & School of Biological and Food Engineering, Changzhou University, Changzhou 213164, China; fanboxiaoyu@cczu.edu.cn (B.F.); s24090860004@smail.cczu.edu.cn (S.F.); tw2022@cczu.edu.cn (W.T.); 2 Changzhou Pharmaceutical Factory Co., Ltd., Changzhou University, Changzhou 213164, China; zhuyijun22@126.com; 3 College of Chemical Engineering, Nanjing Forestry University, Nanjing 210037, China

**Keywords:** helicid analogue, mutation, glycosylation, lignin derivative, biocatalysis

## Abstract

Lignin, as one of the three primary components of renewable lignocellulosic biomass, can be converted into aromatic platform chemicals and holds significant potential for high-value applications. *p*-Hydroxybenzaldehyde is a compound derived from lignin. In this study, the mutant Δ60 of the glycosyltransferase UGT_BL_1 derived from *Bacillus licheniformis* was adopted to catalyze the glycosylation reaction of *p*-hydroxybenzaldehyde, producing a bioactive compound Helicid analogue (*p*-hydroxybenzaldehyde *β*-glucoside). Truncation mutations targeting loop regions may reduce local flexibility, thereby facilitating enhanced access of *p*-hydroxybenzaldehyde to the active site pocket and promoting relative activity. Under optimal conditions (35 °C, pH 7.5, and glucose 200 mM), a high yield of 97.8% for *p*-hydroxybenzaldehyde *β*-glucoside was achieved from 2 mM *p*-hydroxybenzaldehyde within 10 h. The conversion of 3 mM *p*-hydroxybenzaldehyde (366.4 mg/L) yielded up to 2.7 mM (767.5 mg/L) of *p*-hydroxybenzaldehyde *β*-glucoside within 48 h. According to the molecular docking results, the CDOCKER energy value of mutant Δ60 was lower than that of the wild-type, at −16.0 kcal/mol. To our knowledge, this is the first example of an efficient and environmentally sustainable approach for the synthesis of *p*-hydroxybenzaldehyde *β*-glucoside, providing a new insight for the valorization of lignin into valuable biobased chemicals.

## Simple Summary

This study enhanced the relative activity of glycosyltransferase UGT_BL_1 through engineering mutations near its loop regions. The engineered enzyme efficiently catalyzed the glycosylation of the lignin-derived compound *p*-hydroxybenzaldehyde (C_7_H_6_O_2_), enabling the environmentally benign synthesis of a Helicid analogue (*p*-hydroxybenzaldehyde *β*-glucoside (C_13_H_16_O_7_)). Accordingly, this work provided novel insights for lignin valorization. Under optimized reaction conditions (35 °C, pH 7.5, 200 mM glucose (C_6_H_12_O_6_)), an exceptional yield of 97.8% for *p*-hydroxybenzaldehyde *β*-glucoside was attained within 10 h using 2 mM *p*-hydroxybenzaldehyde substrate. Biotransformation of 3 mM *p*-hydroxybenzaldehyde (366.4 mg/L) afforded up to 2.7 mM of target *β*-glucoside product (767.5 mg/L). This research establishes a simpler, more economical, and sustainable enzymatic approach for the efficient synthesis of *p*-hydroxybenzaldehyde *β*-glucoside, offering significant potential benefits for pharmaceutical, cosmetic, or agricultural applications.

## 1. Introduction

Currently, as industrial development progresses, fossil resources are becoming increasingly scarce. Consequently, the utilization of biomass as a substitute for fossil resources has emerged as a promising and sustainable approach. Among various biomass sources, lignocellulose, which is one of the most abundant renewable resources worldwide, showcases considerable potential for application in this field [1,2,3]. It is primarily composed of cellulose, hemicellulose, and lignin. To date, both cellulose and hemicellulose have been extensively researched and utilized and can be efficiently transformed into higher-value products. For instance, Wu et al. conducted enzymolysis of cellulose in the pretreated sugarcane bagasse to produce glucose, which was subsequently used in the fermentation process for the production of adipic acid, a key monomer in nylon synthesis [4]. Yang et al. successfully converted hemicellulose derived from *Phyllostachys edulis* into the platform chemical furfural and functional xylo-oligosaccharides using solid acid catalysis [5]. Lignin can be converted into bio-based aromatic platform chemicals containing benzene rings, such as vanillin [6], *p*-hydroxybenzaldehyde [7,8], and syringaldehyde [9]. Furthermore, they can be further converted into various high-value-added compounds. For example, vanillin can undergo transamination catalyzed by transaminase to synthesize vanillyl amine, an important intermediate in the production of capsaicin [10]. Nevertheless, the high-value-added utilization of lignin-derived compounds still requires further development. *p*-Hydroxybenzaldehyde, as one of the lignin derivatives, possesses a wide range of application potentials. For instance, the biologically active compound 2-arylthiazoline can be synthesized from *p*-hydroxybenzaldehyde via vanillyl alcohol oxidase-catalyzed transformation [11]. Novel AB_3_-type porphyrin derivatives can be synthesized via the condensation of *p*-bromobenzaldehyde and *p*-hydroxybenzaldehyde with pyrrole in propionic acid [12]. In order to achieve the high-value utilization of lignin derivatives, this study aims to synthesize *p*-hydroxybenzaldehyde *β*-glucoside, an analogue of Helicid, from *p*-hydroxybenzaldehyde.

Helicid is a natural compound isolated from the fruit of *Helicia nilagirica* Bedd, and it exhibits therapeutic effects in the treatment of headache, insomnia, and depression [13,14]. Although it can be obtained via extraction, this method is generally considered cumbersome and cost-prohibitive. Helicid and its analogues are typically synthesized using *p*-hydroxybenzaldehyde as the starting substrate. For example, Wen et al. chemically synthesized Helicid analogues and assessed their cholinesterase inhibitory activity [15]. He et al. evaluated the *α*-glucosidase inhibitory activity of *p*-hydroxybenzaldehyde *β*-glucoside [16]. *p*-Hydroxybenzaldehyde *β*-glucoside demonstrates superior tyrosinase inhibitory activity compared to *p*-hydroxybenzaldehyde and even the well-known inhibitor arbutin [17]. The conventional chemical synthesis method for the Helicid analogue, *p*-hydroxybenzaldehyde *β*-glucoside, typically involves the use of *p*-hydroxybenzaldehyde and bromo-tetraacetyl glucose as starting materials [17]. In this reaction, the hydroxyl groups of glucose must be protected to yield bromo-tetraacetyl glucose, which subsequently undergoes glycosylation with *p*-hydroxybenzaldehyde followed by deprotection. This synthetic process entails multiple steps and involves the use of environmentally hazardous catalysts and solvents.

**Figure 1:**
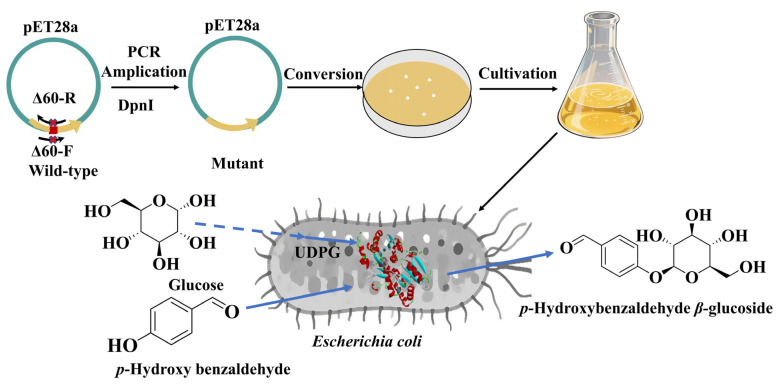
Glucosylation of *p*-hydroxybenzaldehyde for the synthesis of the Helicid analogue.

Biocatalytic synthesis represents an efficient and environmentally sustainable strategy for the production of glycosides [18,19]. Glycosyltransferase exhibits stereoselectivity, mild reaction conditions, and environmental compatibility. In this study, the glycosyltransferase UGT_BL_1 was utilized to catalyze the glucosylation of *p*-hydroxybenzaldehyde for the synthesis of the Helicid analogue *p*-hydroxybenzaldehyde *β*-glucoside. To enhance its relative activity, truncation mutations were introduced to amino acid residues near the enzyme’s substrate binding pocket (Figure 1). Truncation of low-conservation loop regions may induce subtle conformational adjustments in proximal protein domains, thereby favorably modulating catalytic properties. Following systematic optimization of reaction parameters, an efficient synthetic method for *p*-hydroxybenzaldehyde *β*-glucoside was established based on whole-cell catalysis using the engineered glycosyltransferase mutant.

## 2. Materials and Methods

### 2.1. Materials

The *p*-hydroxybenzaldehyde, potassium dihydrogen phosphate, and potassium hydrogen phosphate were obtained from Leyan (Shanghai, China). Glucose and sodium chloride were sourced from Heowns (Tianjin, China). *p*-Nitrophenol was purchased from the Sinopharm (Shanghai, China). Tryptone and yeast extract were acquired from Aladdin (Shanghai, China). UDPG and other reagents were obtained from Titan (Shanghai, China).

The UGT_BL_1 gene (GenBank: KP123426.1) was cloned into the plasmid pET28a with *Xho*I and *Eco*RI restriction sites and was maintained in our laboratory. *Escherichia coli* (*E. coli*) BL21(DE3) was used as the expression host. High-Fidelity PCR Master Mix and DpnI endonuclease were purchased from Adamas (Shanghai, China). Primers were synthesized by GENCEFE Biotech (Wuxi, China). The plasmid miniprep kit was obtained from Tsingke (Beijing, China).

### 2.2. Full-Plasmid PCR Mutagenesis of Glycosyltransferase

The wild-type glycosyltransferase UGT_BL_1 exhibits glycosylation activity toward *p*-hydroxybenzaldehyde, which can be further improved through mutagenesis. Based on the structural model of UGT_BL_1, amino acid residues 59–69 located in the N-terminal substrate binding pocket were selected as mutation targets. Specific primers were designed and synthesized according to the wild-type glycosyltransferase gene sequence (primer sequences are provided in Table S1 (see Supplementary File). Single-point deletions were introduced at each position from 59 (Thr) to 69 (Glu) to generate truncated mutants. The amino acid sequence is listed in Table S2 (see the Supplementary File) This region was selected for modification due to its proximity to the active center, low conservation level, and the presence of loops and *α*-helices. The 25 μL full-plasmid PCR reaction mixture consisted of 0.5 μL template plasmid, 1 μL forward primer, 1 μL reverse primer, 10 μL ddH_2_O, and 12.5 μL High-Fidelity PCR Master Mix. PCR amplification was performed under the following conditions: 30 cycles of denaturation at 95 °C for 30 s, annealing at 62 °C for 30 s, and extension at 72 °C for 4 min. Following amplification, the PCR products were treated with DpnI endonuclease to degrade the methylated parental DNA and subsequently transformed into *E. coli* BL21(DE3). Positive transformants were identified by colony PCR using T7 universal primers and further confirmed by DNA sequencing at GENCEFE Biotech.

### 2.3. Cultivation of Engineered Bacteria and Enzyme Expression

Engineered *E. coli* strains were inoculated onto LB agar plates supplemented with 50 μg/mL kanamycin and incubated overnight at 37 °C. A single colony was selected and transferred into 5 mL of LB liquid medium containing 50 μg/mL kanamycin, followed by incubation at 37 °C and 180 rpm overnight to generate the seed culture. The seed culture was then inoculated into fresh LB liquid medium (at a 5% *v*/*v* ratio) containing 50 μg/mL kanamycin and cultured under the same conditions until the OD_600_ reached 0.6–0.8. Protein expression was induced by the addition of 0.5 mM IPTG, and the culture was further incubated at 25 °C for 12–16 h. Following induction, the bacterial cells were harvested by centrifugation at 8000 rpm for 3 min.

### 2.4. Optimization of Whole-Cell Reaction Conditions

Whole-cell catalysis represents an environmentally benign alternative to traditional chemical catalysts [20]. The typical whole-cell catalytic reaction was conducted under the following conditions: 2 mM *p*-hydroxybenzaldehyde, whole cell (0.025 g/mL), 100 mM glucose, 360 μL buffer solution, pH 7.0, 30 °C, and 180 rpm. The reaction was terminated by adding an equal volume of methanol to the reaction mixture. Subsequently, the samples were centrifuged at 4300× *g* for 3 min, and the supernatant was filtered through a 0.22 μm membrane filter prior to HPLC analysis. To improve the efficiency of whole-cell catalysis mediated by the glycosyltransferase, the effects of reaction time, substrate concentration, glucose concentration from 0–350 mM, reaction temperature in the range of 25 to 50 °C, solution pH between 6.0 and 8.5, and Δ60 loading from 0.01–0.045 g/mL were systematically evaluated. The formula for calculating relative activity is given as below:

(1) $$Relative\;activity\left(\%\right)=\frac{A_{sample}}{A_{control}}\times 100$$

### 2.5. Homology Modeling and Molecular Docking Analysis

Homology modeling based on amino acid sequences was performed using the Swiss-Model server to predict the 3D structures of glycosyltransferase UGT_BL_1 wild-type and its mutant Δ60. The glycosyltransferase (PDB:7VLB) from *Bacillus spizizenii* was selected as the template for modeling UGT_BL_1 from *Bacillus licheniformis*, which shares a sequence identity of 56.80%. The 3D structure of *p*-hydroxybenzaldehyde was imported into Discovery Studio 2019 and subjected to molecular structure optimization. Molecular docking simulations between *p*-hydroxybenzaldehyde and both the wild-type and mutant Δ60 were performed using the CDOCKER module in Discovery Studio 2019 Client. The interactions between the enzyme and the substrate were analyzed in terms of intermolecular binding modes and CDOCKER interaction energies [21]. The settlement formula for binding energy is as below:

EnergyBinding = EnergyComplex − EnergyLigand − EnergyReceptor (2)

### 2.6. HPLC and High-Resolution Mass Spectrometry Analysis Methods

The Thermo Fisher Vanquish Core HPLC system (Germering, Germany) with Athena C18-WP column (ANPEL Laboratory Technologies (Shanghai) Inc., Shanghai, China) (5 μm, 100 A, 4.6 × 250 mm) was adopted for the detection and analysis of *p*-hydroxybenzaldehyde and *p*-hydroxybenzaldehyde *β*-glucoside. Detection was carried out at 275 nm. The column temperature maintained at 30 °C, and the mobile phase was controlled at a flow rate of 1 mL/min. The mobile phase consisted of water (eluent A) and methanol (eluent B). The gradient elution program was as follows: 0–4 min, 10% B; 4–15 min, 10–50% B; 15–19 min, 50% B; 19–26 min, 50–100% B; 26–31 min, 100% B; 31–33 min, 100–10% B; 33–35 min, 10% B. HRMS data were acquired by analyzing samples using an Agilent 6230 TOF mass spectrometer(Agilent Technologies, Santa Clara, CA, USA).

## 3. Results and Discussion

### 3.1. Construction of the Mutant Library of Glycosyltransferase and Protein Expression

**Figure 2:**
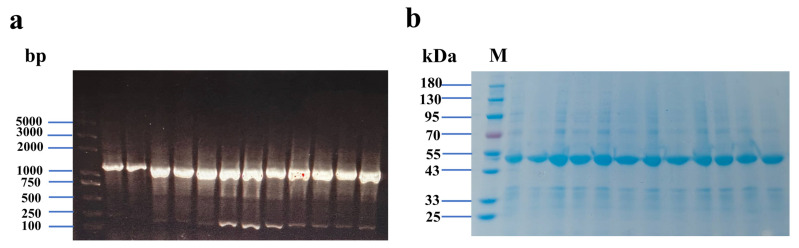
Electrophoretic analysis of gene fragments. Lanes (left to right): marker, wild-type, truncation mutants Δ59 through Δ69 (**a**); SDS-PAGE analysis of protein Lanes (left to right): marker, wild-type, truncation mutants Δ59 through Δ69 (**b**).

Using the plasmid encoding the wild-type glycosyltransferase as a template, the truncated target gene was successfully amplified via whole-plasmid PCR. Fan et al. achieved site-directed mutagenesis of a glycosyltransferase via whole-plasmid PCR, resulting in resveratrol with 87.7% regioselectivity [22]. By modifying the loop region, the channel can be altered, reducing the restraint on the substrate entering the enzyme’s interior and enhancing the enzymatic activity. The nucleic acid electrophoresis results of positive clones obtained through colony PCR are presented in Figure 2a, indicating that the DNA fragment size is approximately 1500 bp, which is consistent with the fragment size between the T7 primer pairs. SDS-PAGE analysis confirmed the successful expression of the mutant glycosyltransferase in *E. coli* BL21(DE3). As shown in Figure 2b, the molecular weight of the expressed protein is close to 49 kDa, and the expression of the mutant is comparable to that of the wild-type. Furthermore, the protein electrophoresis was carried out utilizing the supernatant derived from cell disruption (Figure S1, see Supplementary File).

### 3.2. Screening of Glycosyltransferase Mutants

**Figure 3:**
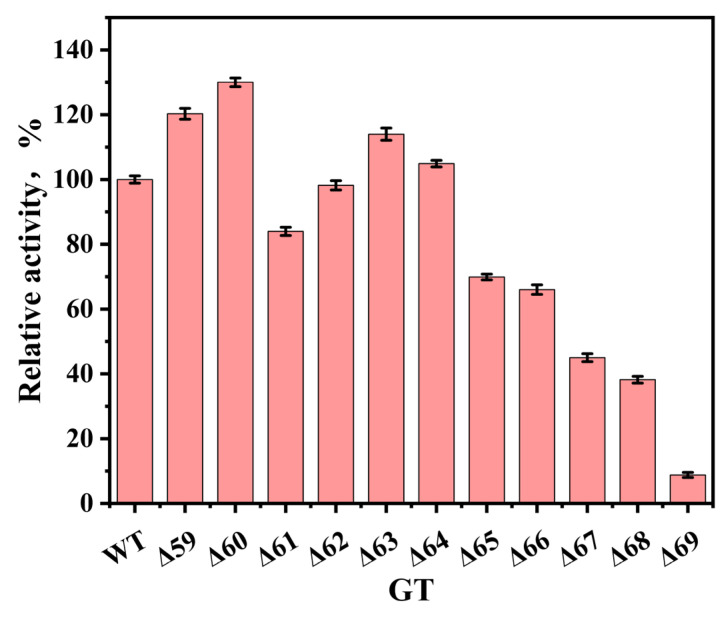
Comparison of glycosylation activity toward *p*-hydroxybenzaldehyde between UGT_BL_1 wild-type and mutants.

By sequentially deleting each amino acid residue from positions 59 to 69 of UGT_BL_1, a total of 11 truncated mutants were generated. Their catalytic efficiencies in glycosylating *p*-hydroxybenzaldehyde were evaluated and compared with that of the wild-type. As illustrated in Figure 3, the mutants Δ59, Δ60, Δ63, and Δ64 exhibited the enhanced catalytic activities relative to the wild-type. Among them, mutant Δ60 displayed the highest relative activity, with an increase of approximately 30% compared to that of the wild-type. Residue 60 is located within the loop, and its deletion resulted in the observed change in enzymatic activity. Wang et al. reported that truncation mutations of *γ*-glutamyltranspeptidase II enhanced the hydrolytic activity in all mutants compared to the wild-type [23]. However, this research revealed that not all truncation mutants exhibited the improved activity, possibly due to the impaired substrate binding caused by loop region modifications. This suggested that not all mutation sites near the loop region are suitable for engineering. Based on above results, a whole-cell catalytic system was established using the engineered strain expressing the mutant Δ60 and subsequently optimized.

### 3.3. Molecular Docking Results

**Figure 4:**
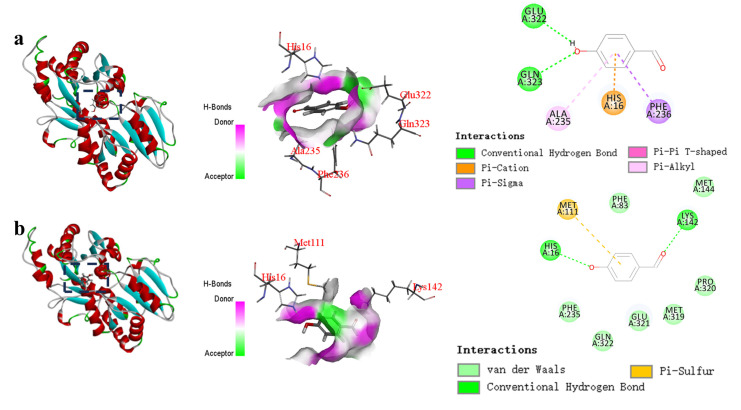
Molecular docking results for wild-type (**a**) and mutant Δ60 (**b**).

Based on the molecular docking analysis of the wild-type glycosyltransferase UGT_BL_1 and *p*-hydroxybenzaldehyde (Figure 4a), the hydrogen-bonds were observed between substrates and residues (GLU322 and GLN323). Additionally, molecular docking reveals that *p*-hydroxybenzaldehyde forms Pi-Alkyl interactions with residues ALA235, and *p*-hydroxybenzaldehyde forms Pi-Cation interactions with residues HIS16, as well as Pi-Sigma interactions with residue PHE236. The docking results indicate that the binding energy between the *p*-hydroxybenzaldehyde molecule and the wild-type is −15.0 kcal/mol.

In the case of the glycosyltransferase mutant Δ60, molecular docking reveals that *p*-hydroxybenzaldehyde forms hydrogen bonds with residues HIS16 and LYS142, as well as a Pi-Sulfur interaction with residue MET111. The calculated binding energy between *p*-hydroxybenzaldehyde and mutant Δ60 is −16.0 kcal/mol (Figure 4b). Notably, a key catalytic residue in the mutant, residue HIS16, can directly interact with *p*-hydroxybenzaldehyde.

The CDOCKER energy, a commonly used metric for evaluating ligand–receptor binding strength, suggests that a higher absolute value corresponds to a stronger binding affinity [24]. A comparative analysis of the CDOCKER energies reveals that the absolute value of binding energy of *p*-hydroxybenzaldehyde with mutant Δ60 is higher than that with the wild-type. The difference in binding energy between the wild type and mutant 60 is 1.0 kcal/mol, indicating a stronger affinity between the mutant and the substrate. The dominant forces governing molecular docking are salt bridges and hydrogen-bonds. The hydrogen-bonds stabilize ligand conformation within the binding pocket through polar interactions, with their strength exhibiting distance-dependent constraints [25]. Notably, the donor-acceptor distance profoundly impacts docking outcomes. In this research, the key residue HIS16 was identified as critical, with mutant Δ60 demonstrating the elevated binding affinity towards *p*-hydroxybenzaldehyde compared to the wild-type enzyme. This improvement might be due to the direct hydrogen-bond interaction between the residue HIS16 and the ligand. A higher binding energy between an enzyme and its substrate indicates a more stable interaction [26]. This enhanced binding affinity may contribute to the higher glycosylation efficiency of the mutant Δ60 toward *p*-hydroxybenzaldehyde compared to the wild-type.

### 3.4. Analysis of the Reaction Products

Based on the HPLC chromatogram of the reaction mixture, showing two distinct peaks (Figure S2, see Supplementary File). The peak observed at a retention time of 17.3 min corresponds to *p*-hydroxybenzaldehyde, which matches the retention time of the standard substrate. The peak, appearing at 11.9 min, was identified as *p*-hydroxybenzaldehyde *β*-glucoside and was further confirmed by high resolution mass spectrometry analysis.

Based on displays the mass spectrum of the substrate (Figure S3, see Supplementary File), *p*-hydroxybenzaldehyde, where the measured mass-to-charge ratio of 123.0449 corresponds to the protonated molecular ion [M+H]^+^, consistent with the theoretical value of 123.0446. Figure S4 (see Supplementary File) illustrates the mass spectrum of the product, *p*-hydroxybenzaldehyde *β*-glucoside, showing a mass-to-charge ratio of 307.0795, which closely matches the theoretical value of the sodium adduct ion [M+Na]^+^ at 307.0794. The molecular weight difference between the two compounds is 162, corresponding to the molecular weight of a single glucose unit (C_6_H_10_O_5_). These results confirm that *p*-hydroxybenzaldehyde was successfully glycosylated to form *p*-hydroxybenzaldehyde *β*-glucoside.

### 3.5. The Effect of Glucose Concentration on the Reaction System

**Figure 5:**
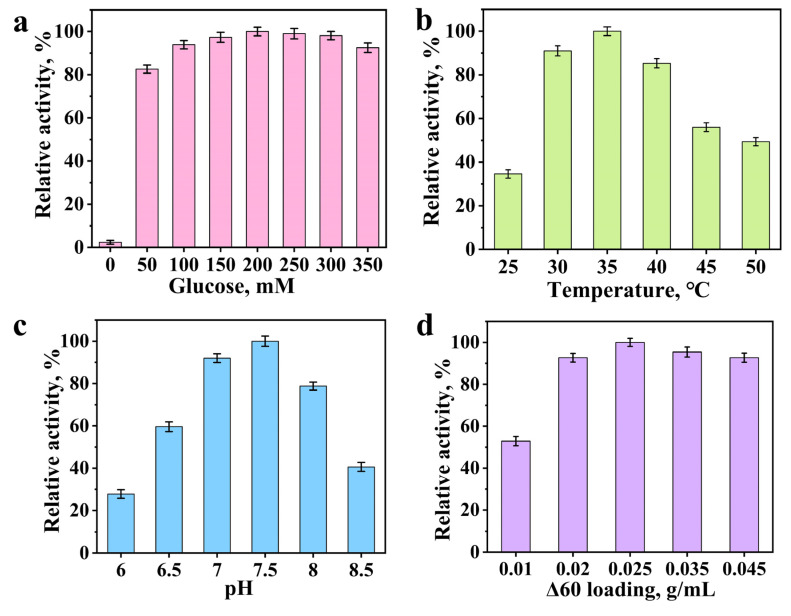
Effect of glucose concentration (0–350 mM) on glycosylation of *p*-hydroxybenzaldehyde catalyzed by mutant Δ60 (35 °C, pH 7.5, 0.025 g/mL, 10 h reaction) (**a**); Effect of temperature (25–50 °C) on glycosylation of *p*-hydroxybenzaldehyde catalyzed by mutant Δ60 (200 mM glucose, pH 7.5, 0.025 g/mL, 10 h reaction) (**b**); Effect of pH (6–8.5) on glycosylation of *p*-hydroxybenzaldehyde catalyzed by mutant Δ60 (200 mM glucose, 35 °C, 0.025 g/mL, 10 h reaction) (**c**); Effect of mutant Δ60 cell loading (0.01–0.045 g/mL) on the glycosylation of *p*-hydroxybenzaldehyde (35 °C, pH 7.5, 200 mM glucose, 10 h reaction) (**d**).

During glycosyltransferase-catalyzed glycosylation reactions, UDPG serves as the glycosyl donor. However, UDPG is relatively costly. To address this issue, glucose was employed as the precursor of UDPG in the whole-cell catalytic system. Glucose can be metabolically converted to UDPG by *E. coli*. The relative activity of the system can be influenced by the concentration of glucose supplied [27]. The impact of glucose concentration on the reaction system is presented in Figure 5a. In the absence of glucose supplementation, the reaction proceeded to a minimal extent, with the trace amount of product likely originating from the basal level of UDPG naturally present in the cells. Upon adding glucose, the relative activity elevated with increasing glucose concentration. Maximum relative activity was observed in the occurrence of 200 mM glucose. However, further elevation in glucose concentration did not result in a continued increase in activity, and a decline was noted when glucose was supplemented in a higher concentration. Excessive glucose supplementary may have interfering effects on the whole-cell system. It seems that the concentration of glucose affects the relative activity of enzymes, with peak performance occurring at the optimal concentration and a progressive reduction in activity as the concentration deviates from this optimum [28]. This phenomenon may be attributed to the increased viscosity of the reaction medium accompanying the elevation in co-substrate concentration, which in turn may impair the activity of the glycosyltransferase [29].

### 3.6. Effects of Reaction Temperature and pH on the Glycosylation System

Enzyme activity is highly dependent on temperature, with distinct variations observed across different temperature ranges [30,31]. As illustrated in Figure 5b, the relative activity of glycosyltransferase in synthesizing *p*-hydroxybenzaldehyde *β*-glucoside was significantly influenced by reaction temperature. Between 25 °C and 35 °C, the relative activity increased with rising temperature, reaching its maximum at 35 °C. Upon raising temperature from 35 °C to 50 °C, a progressive decline in activity was observed. Over 35 °C, the elevated temperatures might lead to a marked reduction in catalytic performance. Beyond the optimal temperature, further temperature elevation may induce a progressive decline in bioreaction rate, as excessively high temperatures can trigger protein denaturation and subsequent structural alterations that lead to irreversible enzyme deactivation [32]. Consequently, 35 °C was the optimal temperature for the bioreaction.

The pH of bioreaction system significantly influences the glycosyltransferase activity. As illustrated in Figure 5c, the glycosylation activity gradually increased as the pH rose from 6.0 to 7.5, reaching maximum activity at pH 7.5. Beyond this optimal point, further increasing pH led to a progressive inhibition of enzymatic activity [33,34]. At pH 7.5, the glycosylation reaction activity began to be inhibited. Upon raising the pH from 7.5 to 8.5, the relative activity progressively declined. It can be concluded that the optimal pH for the bioreaction was 7.5.

### 3.7. The Impact of Bacterial Load on Reaction Outcomes

The cells loading could significantly influence the product yield of the relative activity (Figure 5d). Under the reaction temperature of 35 °C and pH 7.5, the relative activity was evaluated across varying bacterial concentrations in the presence of 2 mM *p*-hydroxybenzaldehyde (as substrate) and 200 mM glucose. The glycosylation activity elevated gradually as the bacterial cell loading was raised from 0.01 g/mL to 0.025 g/mL, reaching its maximum at 0.025 g/mL, followed by a gradual decline at higher loads. The enzyme exhibited the maximal relative activity when the bacterial concentration was 0.025 g/mL. This concentration represents the optimal bacterial concentration for the reaction. Any deviation from this optimal level might result in the reduced enzymatic activity [35,36,37]. It can be concluded that 0.025 g/mL represented the optimal bacterial concentration for the reaction.

### 3.8. Effects of Substrate Concentration on Biocatalytic Activity

**Figure 6:**
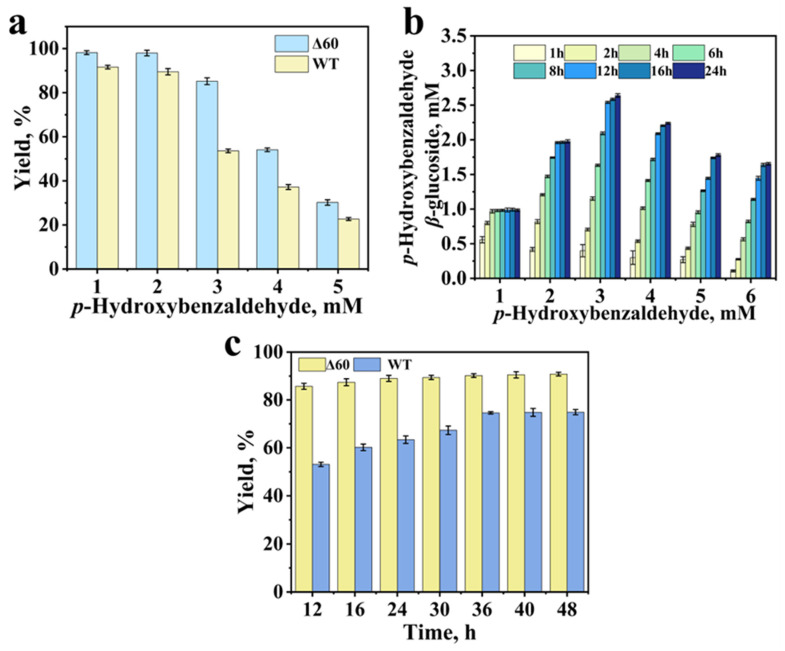
Effect of substrate concentration on the catalytic synthesis of *p*-hydroxybenzaldehyde *β*-glucoside by wild-type and mutant Δ60 (12 h) (35 °C, pH 7.5, 200 mM glucose, 0.025 g/mL cell loading) (**a**); Time course of *p*-hydroxybenzaldehyde *β*-glucoside synthesis by mutant Δ60 at different substrate concentrations (0–24 h) (35 °C, pH 7.5, 200 mM glucose, 0.025 g/mL mutant Δ60 cell) (**b**); Time-dependent production of *p*-hydroxybenzaldehyde *β*-glucoside by wild-type and mutant Δ60 at 3 mM *p*-hydroxybenzaldehyde (0–48 h) (35 °C, pH 7.5, 200 mM glucose, 0.025 g/mL cell loading) (**c**).

The effect of substrate concentration on the reaction is evidenced in Figure 6a, depicting the reaction progress after 12 h for both the wild-type enzyme and the mutant Δ60. The impact of substrate concentration on the reaction is shown in Figure 6a, which presents the progress after 12 h of reaction. The activity of mutant Δ60 was higher than that of the wild type throughout. Notably, at a substrate concentration of 3 mM, the yield decreased significantly. As the substrate concentration further increased, the rate of the glycosylation reaction began to decline. Consequently, with increasing substrate loading, the yield progressively dropped at the same reaction time point (12 h). At high substrate concentrations, the reaction velocity decreased. This behavior indicated that the substrate inhibition occurred. The inhibition may arise because high substrate dose can disrupt the enzyme’s normal catalytic capacity, impairing its ability to catalyze the reaction efficiently [38]. Alternatively, it is possible that *p*-hydroxybenzaldehyde inhibited the activity of *E. coli* cells (or the enzyme within cell). As the substrate dose increased, this inhibitory effect would consequently weaken the relative activity [39]. A time-course experiment was conducted on mutant Δ60 (Figure 6b), and the results indicated that the response had not yet reached its maximum value before 24 h.

A time-course experiment was implemented for converting 3 mM substrate (Figure 6c). The reaction essentially reached the maximum product concentration after 24 h. Figure 6a presents a comparison of the progress of the mutant and the wild type after 12 h of reaction, indicating that the substrate concentration has a significant impact on the reaction activity. Under the catalysis by Δ60, the accumulated maximum product concentration reached 2.7 mM.

Biomass represents an abundant and renewable biological resource primarily composed of cellulose, hemicellulose, and lignin [40,41,42]. While cellulose and hemicellulose have been extensively utilized [43,44,45], the valorization of lignin-derived compounds remains underdeveloped [46,47]. This study employed *p*-hydroxybenzaldehyde, a representative lignin derivative, as a model substrate to advance novel lignin valorization strategies. The biocatalytic transformation of *p*-hydroxybenzaldehyde into *p*-hydroxybenzaldehyde *β*-glucoside was implemented, simultaneously addressing limitations of traditional extraction methods and reducing environmental impacts inherent to chemical synthesis. Through the targeted mutagenesis of wild-type glycosyltransferase, the catalytic efficacy was enhanced, resulting in the accelerated glycosylation kinetics. Notably, substituting costly uridine diphosphate glucose (UDPG) with glucose as glycosyl donor can significantly reduce the production costs. Under optimized conditions, 2 mM *p*-hydroxybenzaldehyde was converted to the target product within 10 h with a yield of 97.8%. Furthermore, when the substrate concentration was increased to 3 mM, 2.7 mM (767.5 mg/L) of the product was obtained.

Further research efforts, guided by the results of this study, should leverage more systematic approaches to mutagenesis for advanced strain development. By strategically screening mutation sites to enhance substrate tolerance, a breakthrough increase in product yield and a simultaneous improvement in substrate loading capacity can ultimately be achieved [48,49,50]. Utilizing appropriate mutation strategy to significantly increase *p*-hydroxybenzaldehyde *β*-glucoside titers while maintaining relative activity, thereby advancing industrial feasibility of this bioprocess. The developed glycosylation methodology demonstrates both economic viability and operational robustness. Furthermore, expanding the substrate scope to include diverse aldehydes presents promising exploratory avenues. In a concise summary, this study establishes a green and efficient biocatalytic route for synthesizing *p*-hydroxybenzaldehyde *β*-glucoside while enabling high-value biomass utilization. To facilitate industrial-scale production, subsequent efforts should focus on reducing raw material costs and enhancing the relative activity of the enzyme through rational strain engineering.

## 4. Conclusions

The glycosyltransferase UGT_BL_1 was engineered via truncation mutation, yielding the mutant designated as Δ60, which exhibited the highest activity towards *p*-hydroxybenzaldehyde among the screened variants. A whole-cell catalytic system was subsequently developed to efficiently convert *p*-hydroxybenzaldehyde into *p*-hydroxybenzaldehyde *β*-glucoside. To enhance the economic feasibility of the process, glucose was employed as an inexpensive glycosyl donor substitute for costly UDPG. Under optimized reaction conditions, a maximum product concentration of 2.7 mM (767.5 mg/L) could be achieved. There is limited information about the efficient transformation of *p*-hydroxybenzaldehyde into valuable *p*-hydroxybenzaldehyde *β*-glucoside. The truncated mutation method employed in this study could enhance the relative activity of glycosyltransferase, with a concentration of 2 mM of *p*-hydroxybenzaldehyde, the yield could reach 97.8% within 10 h, providing an eco-friendlier approach for the efficient synthesis of *p*-hydroxybenzaldehyde *β*-glucoside.

## Data Availability

The original contributions presented in this study are included in the article/Supplementary Materials. Further inquiries can be directed to the corresponding author.

## Supplementary Materials

The following supporting information can be downloaded at: https://www.mdpi.com/article/10.3390/biology14101358/s1. Figure S1: Electrophoresis profile of supernatant protein from cell lysate. Figure S2: HPLC chromatogram. Figure S3: HRMS spectrum of the substrate *p*-hydroxybenzaldehyde. Figure S4: HRMS spectrum of the product *p*-hydroxybenzaldehyde *β*-glucoside. Table S1: Primers employed in this study. Table S2: The amino acid sequence.
