# Supplementary material for: Efficient Bioproduction of p-Hydroxybenzaldehyde β-Glucoside from p-Hydroxybenzaldehyde by Glycosyltransferase Mutant UGTBL1-Δ60
Source: Biology (Basel). 2025 Oct 3;14(10):1358. doi: 10.3390/biology14101358 (PMC12561349; doi:10.3390/biology14101358)
Supplement: Supplementary file 1 [file biology-14-01358-s001.zip › biology-3842738-supplementary.pdf]

Supplementary Materials

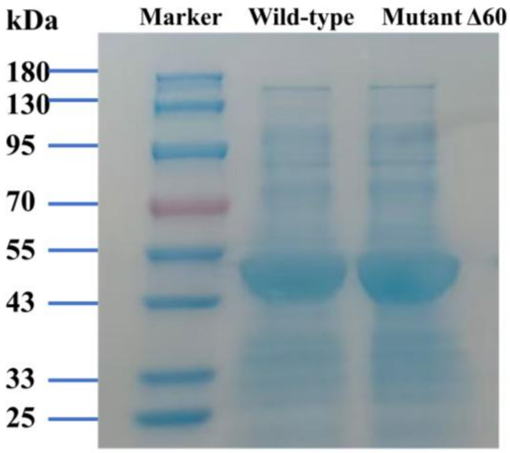

Figure S1. Electrophoresis profile of supernatant protein from cell lysate.

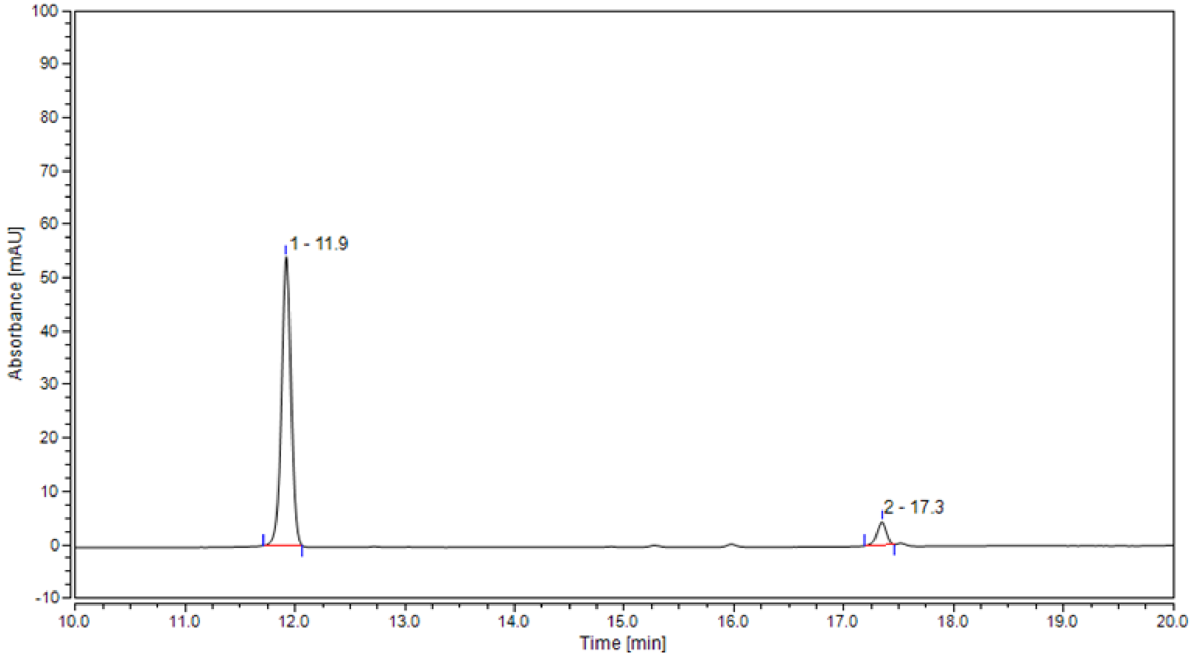

Figure S2. HPLC chromatogram (the peak eluting at 11.9 min corresponds to *p*-hydroxybenzaldehyde *β*-glucoside, while the peak with a retention time of 17.3 min is identified as *p*-hydroxybenzaldehyde).

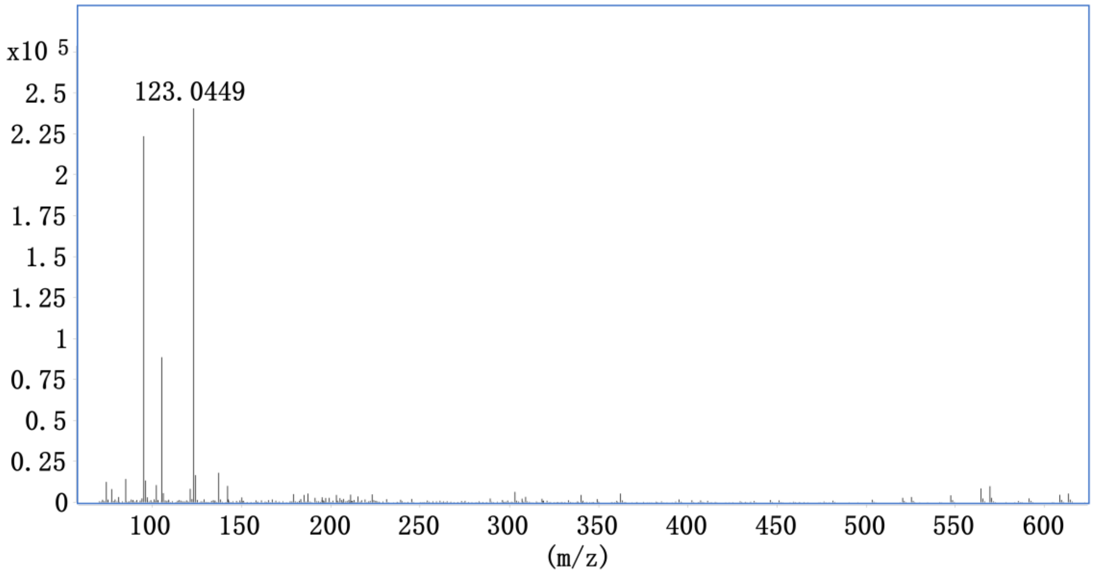

Figure S3. HRMS spectrum of the substrate *p*-hydroxybenzaldehyde.

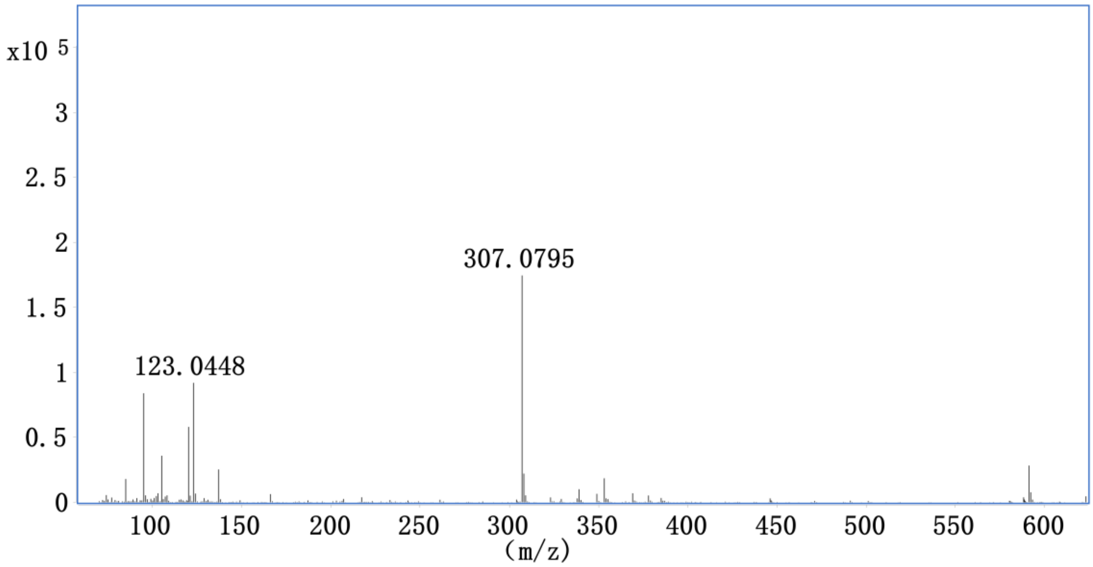

Figure S4. HRMS spectrum of the product *p*-hydroxybenzaldehyde *β*-glucoside.

Table S1. Primers employed in this study.

Primer	Sequence (5' to 3')
Δ59-F	5'- GCTCAACTACCGCTCATTAAATATCGATCCGCAGCAAA -3'
Δ59-R	5'- AATGAGCGGTAGTTGAGCGGCTCTGCCCCAGTT -3'
Δ60-F	5'- CTAATATCGATCCGCAGCAAATTCGGGAGCTGA -3'
Δ60-R	5'- GCTGCGGATCGATATTAGTTGAGCGGTAGTTGAGCGG -3'
Δ61-F	5'- CTTTAATCGATCCGCAGCAAATTCGGGAGCTGA -3'
Δ61-R	5'- GCTGCGGATCGATTAAAGTTGAGCGGTAGTTGAGCG -3'
Δ62-F	5'- TGATCCGCAGCAAATTCGGGAGCTGATGAAAAA -3'
Δ62-R	5'- CGAATTTGCTGCGGATCATTTAAAGTTGAGCGGTAGTTGAGC -3'
Δ63-F	5'- TATCCCGCAGCAAATTCGGGAGCTGATGAAAAA -3'
Δ63-R	5'- CGAATTTGCTGCGGGATATTTAAAGTTGAGCGGTAGTTGAG -3'
Δ64-F	5'- TCGATCAGCAAATTCGGGAGCTGATGAAAAATA -3'
Δ64-R	5'- CCCGAATTTGCTGATCGATATTTAAAGTTGAGCGGTAGT -3'
Δ65-F	5'- ATCGATCCGCAAATTCGGGAGCTGATGAAAAAT -3'
Δ65-R	5'- CCGAATTTGCGGATCGATATTTAAAGTTGAGCG -3'
Δ66-F	5'- TATCGATCCGCAGATTCGGGAGCTGATGAAAAATAA -3'
Δ66-R	5'- CGAATCTGCGGATCGATATTTAAAGTTGAGCGG -3'
Δ67-F	5'- CAACGGGAGCTGATGAAAAATAAAAAGGATATG -3'
Δ67-R	5'- TTTCATCAGCTCCCGTTGCTGCGGATCGATATTTAAA -3'
Δ68-F	5'- CGCAGCAAATTGAGCTGATGAAAAATAAAAAGGATATG -3'
Δ68-R	5'- TCAGCTCAATTTGCTGCGGATCGATATTTAAAG -3'
Δ69-F	5'- CAGCAAATTCGGCTGATGAAAAATAAAAAGGATATGACAC -3'
Δ69-R	5'- CATCAGCCGAATTTGCTGCGGATCGATATTTAA -3'
T7-20	5'- TAATACGACTCACTATAGG -3'
T7ter	5'- TGCTAGTTATTGCTCAGCGG -3'

Table S2. The amino acid sequence.

MGHKHIAIFNIPAHGHINPTLALTASLVKRGYRVTYPVTDEFVKAVEETGAE PLNYRSTLNIDPQQIRELMKNKKDMTQAPMMFMKEMEEVLPQLEALYENDK PDLILFDFMAMAGKMLAEKFGIEAVRLCSTYAQNEHFSFKSMSEEFKIELTPEQ EAALKNANLPSFNFEEMFEPAKLNIVFMPRAFQPYGETFDERFSFVGPSLAKR KFQEKDTPVISDSGRPVMLISLGTAFNAWPEFYHMCIEAFRDTKWQVIMAVGT TIDPESFDDIPDNFSIHQRVPQLEILKKAELFITHGGMNSTMEGLNAGVPLVAVP QMPEQEITARRVEELGLGKHLQPEDTTVASLREAVSQTDGNLDVLKRVKDMQ EHIKQAGGAEKAADEIESFLAPAGVK
